# Retinoblastoma genetics screening and clinical management

**DOI:** 10.1186/s12920-021-01034-6

**Published:** 2021-07-22

**Authors:** Himika Gupta, Sivasankar Malaichamy, Ashwin Mallipatna, Sakthivel Murugan, Nallathambi Jeyabalan, Vishnu Suresh Babu, Anuprita Ghosh, Arkasubhra Ghosh, Sam Santhosh, Somasekar Seshagiri, Vedam L. Ramprasad, Govindasamy Kumaramanickavel

**Affiliations:** 1 Narayana Nethralaya, Bangalore, India; 2 Grow Lab, Narayana Nethralaya Foundation, Bangalore, India; 3 Medgenome Labs Ltd, Bangalore, India; 4 Genentech, San Francisco, USA

## Abstract

**Background:**

India accounts for 20% of the global retinoblastoma (RB) burden. However, the existing data on *RB1* gene germline mutations and its influence on clinical decisions is minimally explored.

**Methods:**

Fifty children with RB underwent complete clinical examination and appropriate multidisciplinary management. Screening of germline *RB1* gene mutations was performed through next-generation sequencing and Multiplex Ligation-dependent Probe Amplification (MLPA) analysis. The mutation and non-mutation groups were compared for clinical parameters especially severity, progression and recurrence.

**Results:**

Twenty-nine patients had bilateral RB (BLRB) and 21 had unilateral RB (ULRB). The genetic analysis revealed 20 *RB1* variations in 29 probands, inclusive of 3 novel mutations, known 16 mutations and heterozygous whole gene deletions. The mutation detection rate (MDR) was 86.2% in BLRB and 19% in ULRB. Associations of disease recurrence (*p* = 0.021), progression (*p* = 0.000) and higher percentage of optic nerve invasion, subretinal seeds and high-risk pathological factors were observed in the mutation group. Clinical management was influenced by the presence of germline mutations, particularly while deciding on enucleation, frequency of periodic follow up and radiotherapy.

**Conclusions:**

We identified novel *RB1* mutations, and our mutation detection rate was on par with the previous global studies. In our study, genetic results influenced clinical management and we suggest that it should be an essential and integral component of RB-care in India and elsewhere.

## Background

Retinoblastoma (RB) (OMIM#180200) is the commonest childhood intraocular tumor, with a global estimated annual incidence of 1 in 15,000–20,000 live births [1]. India accounts for the highest global burden having one out of every five RB children with an estimated annual incidence of 1500 RB children [2–4]. RB occurs due to the two-hit hypothesis of Knudson, which is because of loss-of-function of the tumour suppressor *RB1*gene, owing to homozygous allelic mutations, loss of heterozygosity mechanism or gene silencing [5]. *RB1* is a nuclear phosphoprotein, essential for G1/S check point during the cell cycle regulation, while in a dephosphorylated state binds to mitotic agents like E2F, viral particles and other factors, but releases them during mitosis when phosphorylated. *RB1* gene is located on chromosome band 13q14.2, consisting of 27 exons, which encodes a 4.7 kb mRNA. So far, 1748 unique *RB1* variants in 3366 individuals have been identified and summarized in the Leiden Open Variation Database (LOVD) [6]. Most of the *RB1* mutations are unique and found in exon, splicing introns and untranslated regions [5–8]. Interestingly, *RB1* exon deletions are seen not only in RB but also less frequently in breast cancer, osteosarcoma and lung cancer.

Usually, in any given population, there are more children with unilateral RB (ULRB-60%) than bilateral (BLRB-40%) and a clinician has to be noted that a majority of those with BLRB and a small proportion of those with ULRB might have germline *RB1* mutations, who may need genetic screening [2]. Genetic screening could play a vital role in management of RB which could influence various crucial clinical management decisions [7].

Unless genetic testing is available, the minority of unilateral hereditary cases, fail to get the desirable clinical management decisions and frequent clinical surveillance. Hereditary RB tends to be early in onset, bilateral and multifocal, hence needs continuous surveillance for effective management. All cases with mutation, as mentioned earlier, have a lifetime risk for osteosarcoma, soft tissue sarcoma, malignant melanoma or multiple brain tumours. Hence they need lifelong follow-ups, as opposed to sporadic cases, which may not have genetic predisposition. Between 1905 and 2005 about 199 RB survivors were retrospectively analysed for second primary tumours (SPT) and found that 44 of them developed SPT [9]. Any form of radiation for investigation (like X-ray, CT scan) or treatment has to be preferably avoided in all germline cases, due to probable increased risk of second malignancies. Besides North America and Germany, *RB1* mutations have been reported from various populations around the world like, Argentina [10], Brazil [11], China [12, 13], Colombia [14], Ecuador [15], Egypt [16], India [17–21], Iran [22], Israel [23, 24], Italy [25], Korea [26], Netherlands [27], Spain [28, 29], Malaysia [30], Mexico [31], Morocco [32], New Zealand [33], Pakistan [34], Swiss [35], Tunisia [36] Singapore [37] Thailand [38] and United Kingdom [39]. Out of five earlier studies from India, stratifying genetic tests is an option suggested by Thirumalairaj et al. [40].

Though enormous number of studies are available on *RB1* gene mutations across the globe, including India, there is limited information on how the genetic result could influence clinical management outcomes. Hence, we undertook this study to describe and correlate the genetic and clinical parameters of 50 RB patients from India. We also examined the opportunities and challenges in clinical decisions which were influenced through *RB1* gene screening in a developing country scenario.

## Methods

### Patient recruitment and clinical examination

Fifty (48 unrelated and two related siblings) RB patients (aged 0.2–5.3 years) with various clinical presentations, from the Department of Paediatric Ophthalmology, Narayana Nethralaya, Bangalore, India were recruited from June 2014 to Feb 2015. Among these twenty-nine were BLRB and twenty-one had ULRB. A complete clinical examination was carried out under general anaesthesia which included dilated retinal evaluation, imaging of retina using wide field fundus camera (Retcam), measurement of intraocular pressure, anterior segment evaluation by handheld slit lamp. Also, magnetic resonance imaging (MRI) of the orbits and brain, B scan ultrasonography of the eye, cerebrospinal fluid analysis and bone marrow analysis were performed when indicated. The clinical disease was classified as per the AJCC TNM classification for RB, as well as the International Classification of Intraocular Retinoblastoma [41]. The study was approved by the Institutional Ethical Committee, which followed the Tenets of the Declaration of Helsinki. After ascertaining pedigree and written informed parental consent, five ml of blood sample was obtained in EDTA coated vacutainer tubes from patients (during examination under anaesthesia) for genetic analysis. For clinical analysis the cohort was divided into two groups—those with and without *RB1* mutations.

### DNA isolation, NGS target sequencing of RB1 gene analysis

Nucleospin Blood XL kit (Macherey–Nagel)—About 5 of peripheral blood from the child is collected and 500 μl of proteinase K and PBS are added for lysis of RBCs. Then 10 ml of buffer BQ1 is added a shaken vigorously for 2 min and incubated at 56 °C for 15 min. Then add 10 ml of 96–100% ethanol and vortex for 10 s for lysate formation. Take Nucleospin Blood XL column and add 15 ml of the lysate and centrifuge at 5000 rpm for 3 min. Discard the flow-through and repeat the last step. Add 7.5 ml Buffer BQ2 and centrifuge at 5000 rpm for 2 min and repeat the step for 20 min. Insert the column into new collection tube and add 750 μl of prewarmed 70 °C Elution Buffer BE and incubate at room temperature for 16 h. The last step may be repeated and when centrifuged at 5000 rpm for 5 min, highly pure genomic DNA elutes through the silica membrane.

Genomic DNA was used for targeted gene capture using a custom capture kit. Briefly, 1ug of DNA was subjected to fragmentation resulting in an average size of 150 bp followed by end repair, adenylation, adaptor ligation and amplification to obtain whole genome libraries using the Kapa DNA library preparation kit v2.14. These libraries were then hybridized to biotinylated probes (NimbleGen, Roche) specific to *RB1*gene for 72 h and extracted using streptavidin beads, washed and normalized. The libraries were then sequenced to mean > 80–100× coverage on Illumina sequencing platform (HiSeq 2500). The sequences obtained are aligned to human reference genome (GRCh37/hg19) using BWA program [42, 43] and analyzed using Picard and GATK-Lite tool kit [44, 45] to identify variants relevant to the clinical indication. Annotations of the variants were performed against the Ensembl release 75 gene model [46]. Clinically relevant mutations were annotated using published variants in literature and a set of variant databases including ClinVar, OMIM, GWAS, HGMD and SwissVar [47–54].

### Multiplex ligation-dependent probe amplification (MLPA) analysis

In order to detect large deletions/duplications in the *RB1* gene, we performed Multiplex Ligation-dependent Probe Amplification (MLPA). SALSA MLPA kit P047 *RB1* (Amsterdam, Netherlands) was used as per manufacturer’s recommendations.

### Statistical analysis

Multivariate analysis for genotype phenotype correlation was done using Pearson Chi square test, SPSS software. Clinical factors like sub retinal seeds, optic nerve invasion, pathological high-risk factors (HRF), tumour recurrence, tumor resistance to treatment, need for 2nd line drugs like topotecan and need for radiotherapy were analysed in the mutation versus no mutation groups.

## Results

**Fig. 1:**
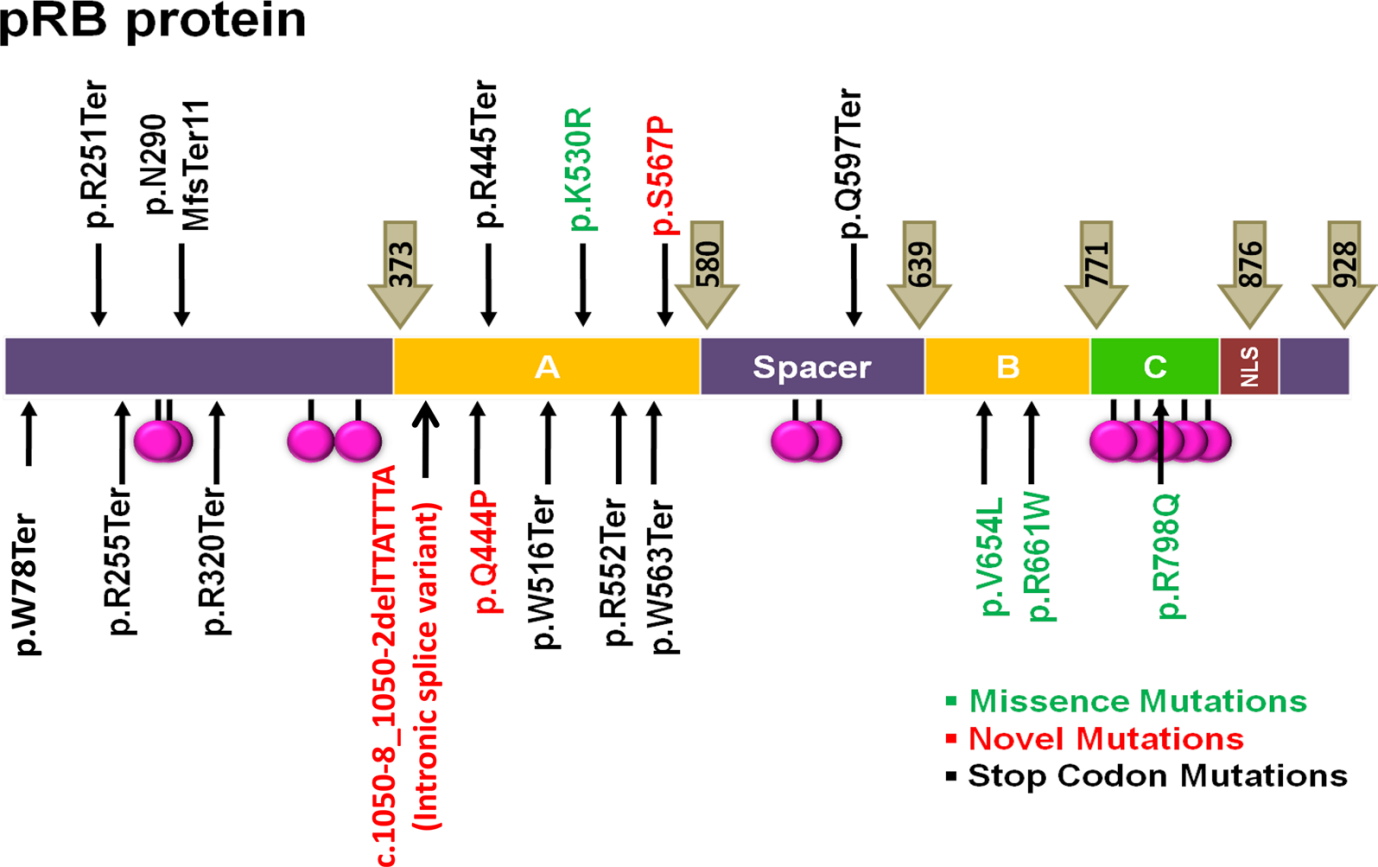
Represents the mutations identified in RB patients distributed across the RB protein structure

Of 50 RB patients, 29 had BLRB (average age at presentation of 1.8 years) and 21 had ULRB (average age at presentation of 2.3 years). A family history of RB was observed in two patients. In the BLRB group, 25 out of 29 probands (86.2%) had a germline mutation whereas in the ULRB group, 4 out of 21 (19%) had a mutation. NGS and MLPA analyses revealed total of 20 *RB1* gene variations in 29 probands, inclusive of three novel mutations (3 probands, 6%—c.1050-8_1050-2delTTATTTA) (intronic splice variant) (ClinVar ID: SCV001571344.1), Q444P (ClinVar ID: SCV001571345.1) and S567P (ClinVar ID: SCV001571346.1)), previously reported 16 mutations (22 probands: 44%) and heterozygous deletion of whole *RB1* gene (3 BLRB, 1 ULRB, 8%). The types of mutations were, non-sense being the maximum [13], followed by missense [7], splice site [4], whole gene deletions [4]. One proband had frameshift (Table 1; Fig. 1).

**Table 1:** Type of genetic abnormalities

S. nos.	Genetic abnormality	Number of mutations	Number of patients	Component of novel mutation	No of pts with novel mutation	Unilateral /bilateral
1	Whole gene deletion	NA	4	NA	NA	1:3
2	Missense mutation	7	7	2	2	3:4
3	Frame shift	1	1	NIL	NIL	0:1
4	Splice site	3	4	1	1	0:4
5	Non-SENSE	10	13	NIL	NIL	0:13
Total		19	29	3	7	4:25

Genotype to clinical analysis revealed that there was no direct correlation between age of presentation and disease severity between the groups. Clinical features, age of presentation and high-risk features like optic nerve invasion in the groups have been listed in Table 2. Mutation group had more patients with increased severity requiring enucleation (95.23%), optic nerve invasion (64.7%), sub-retinal seeds (68%) and pathological high-risk factors (73.9%). The disease severity factors like average clinical TNM and pathological TNM were stratified as per the mutation type (splice site, missense, termination and whole gene deletion) and the findings are listed in Table 3. In the current cohort, splice site mutation had the highest average clinical and pathological TNM, as well as the youngest average age of enucleation. Disease recurrence and disease progression correlated significantly with mutation group (*p* = 0.021 and *p* = 0.000 respectively). Notably, of the total 10 recurrences in the current cohort, 9 patients had the mutation (Table 4). The mutation detection rate (MDR) was 86.2% in BLRB (25 out of 29) and 19% in ULRB (4 out of 21), which was better than many other global studies and comparable to some of the recent robust ones (Table 5).

**Table 2:** Clinical presentation

	Mutation	No mutation
Average age of diagnosis	1.82 years	2.08 years
Need for enucleation	23 of 29(79.31%)	20 of 21(95.23%)
Average age of enucleation	2.08 years*	2.05 years #
Optic nerve invasion	11 of 17 (64.7%)	8 of 19(42.1%)
Sub retinal seeds	17 OF 25(68%)	4 OF 20(20%)
Pathological high risk factors	17 of 23(73.9%)	9 of 19 (47.36%)

*Average age of enucleation calculated after excluding 1 patient who was enucleated at 13.77 years

^#^Includes one pt who presented at 5.13 years and was not enucleated. BL detected early and therefore managed early compared to UL which is late

**Table 3:** Correlating mutation versus clinical disease severity

	Splice site mutation	Missenese mutation	Termination	Whole gene deletion
Total	4	7	14	4
U:B	0:04	3:04	0:14	1:03
Age (years)	1.45	1.81	1.28	4.09
Enucleation	4 (100%)	7 (100%)	10 (71.4%)	3 (75%)
Average age at enucleation (years)	1.83	1.95	2.02	1.98 *
Avg pathological TNM	2.6	2.14	1.85	2.3
Avg clinical TNM	3.3	2.7	2.38	2.75

*Average age of enucleation calculated after excluding 1 patient who was enucleated at 13.77 years

**Table 4:** Correlation of genotype with high risk phenotype

Phenotypic features	Mutation present *n* (%)	No mutation *n* (%)	Pearson Chi-square tests	
			Chi sq	Significance (*p* value)
*Optic nerve invasion*				
Y	11	8	1.83	0.575
N	6	11		
*Recurrence*				
Y	9	1	5.25	0.021
N	20*	20		
*Progression*				
Y	16	1	13.79	0.000
N	13	20		
*Need for topotecan*				
Y	7	1	3.40	0.065
N	22	20		
*Need for radiotherapy*				
Y	3	2	0.091	0.923
N	26	19		

*Of the 20 without recurrence and with mutation, 7 had disease progression and 13 had ‘none’

**Table 5:** Mutation detection rates in unilateral and bilateral RB patient groups studies across the globe

S. nos	Author	Country	Type of mutations	Mutation detection rate BLRB (%)	Mutation detection rate ULRB	Year of study
02	Mohd Khalid, M.K., et al	Malaysia	Nonsense, Frame shift, Splice site and De-novo origin	100	25%	2015
05	Grotta et al.	Italy	Point mutations, Frame shift, Large deletions	96.5	22%	2015
09	Chen, Z., et al	USA	Nonsense, Splice, Frameshift	97	18%	2014
07	Price et al	United Kingdom	Point mutation, deletions, missense, splice site mutations	96	9.5%	2014
10	Seo, S.H., et al	Korea	Missense, nonsense, frameshift and splice	94.5	None	2013
11	Ottaviani, D., et al	Argentina	Nonsense, frameshift, missense, deletions	94	–	2013
08	Dommering, C.J., et al	Netherland	Nonsense, frameshift, splice, large indel, missense, chromosomal deletions and promoter	92	10%	2014
01	Frenkal.S*et al*	France	Stop codon, Splice site and large deletions	90	19.8%	2016
15	Macias, M., et al	Mexico	Nonsense, Splice, Frameshift	76.9	34.8%	2008
16	Abouzeid et al	Switzerland	Nonsense, frameshift, missense, deletions	73	10.7%	2007
03	Zhang, L., et al	China	Nonsense, Splice, Frameshift	65	35%	2015
06	Devarajan et al	India	Nonsense, Frame shift, Splice site and Denovo origin	63	37%	2015
04	Kalsoom, S., et al	Pakistan	Null mutation, deletions, missense, splice site mutations	45.7	54.3%	2015
12	Barbosa, R.H., et al	Brasil	Nonsense, Splice, Frameshift	42.2	56.3%	2013
14	Abidi et al.,	Morocco	Duplication, Deletion, Splice, Frameshift	40	None	2011
17	Choy et al	Hong kong & China	Nonsense, Splice, Frameshift	38	19%	2002
13	Ahani et al	Iran	Missense, frameshift and splice site	16.6	18.2%	2013
14	Present study—Himika, Malaichamy, et al	India	Missense, frameshift, gene deletions	86.2	19%	2020

**Fig. 2:**
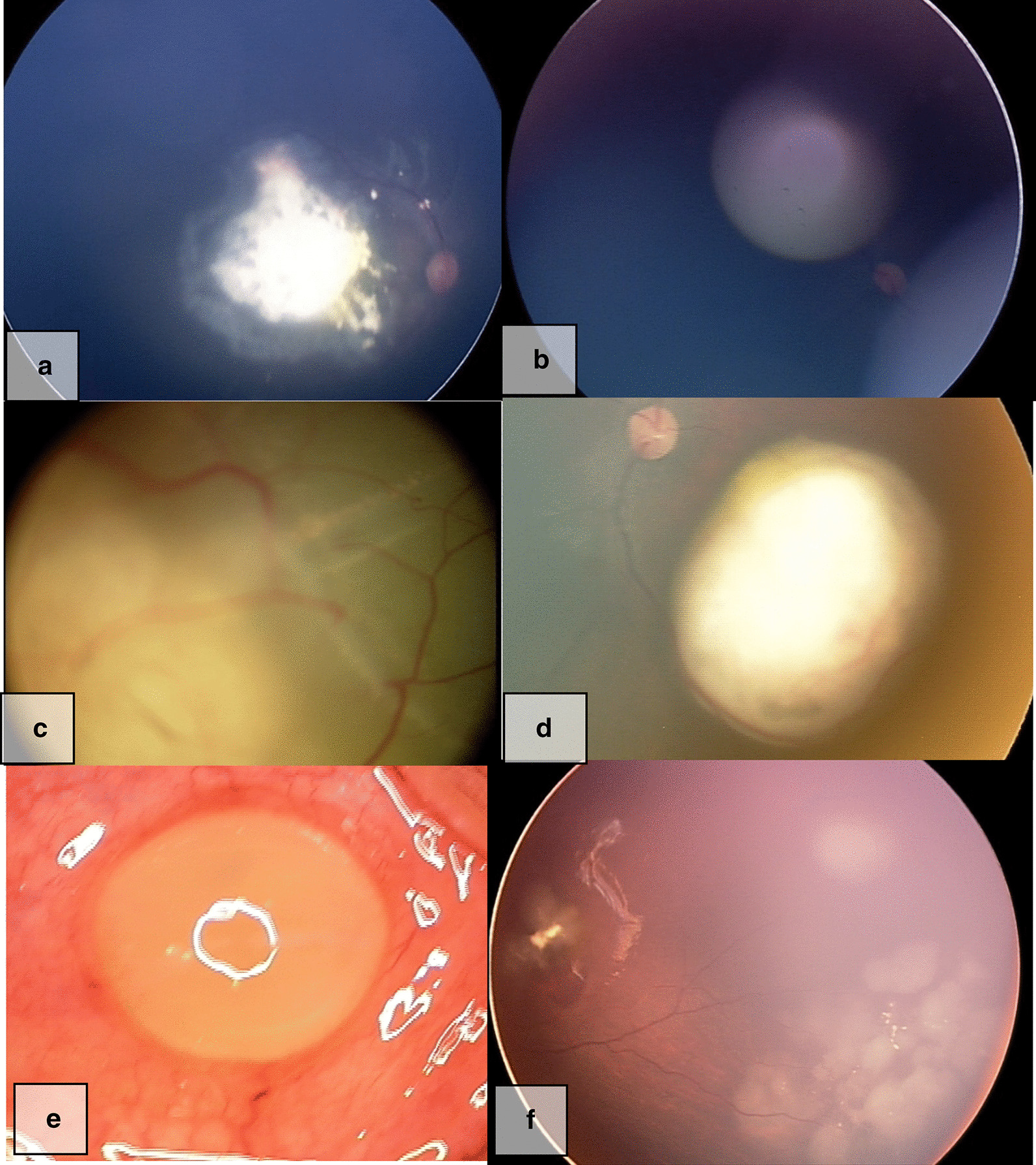
Variable phenotype of same genotype. Case75 **a**, **b** had mild disease in both eyes, bilateral globe salvage successful. Case 55. **c** One eye had severe disease needing enucleation, while other eye **d** had mild disease with successful globe salvage. Case 9 **e** one eye extensive tumor needing enucleation. Later the other eye **f** developed tumor which was nonresponsive to Rx and eventually needed enucleation

A nonsense mutation, c. 233G > A (p.W78Ter) was identified in two unrelated patients with bilateral RB. The novel nucleotide changes include two missense substitutions—c.1699 T > C (p.S567P), c.1331A > C (p.Q444P) and one splice site variation (c.1050-8_1050-2delTTATTTA). Bioinformatics prediction analysis of SIFT, PolyPhen–2, Provean, showed that the missense substitutions (p.S567P, p.Q444P) had deleterious effect which may affect the functional properties of the protein and both the missense variations are present in the retinoblastoma-associated protein A domain of the RB1 protein. The three novel mutations were in BLRB patients. One of the BLRB patients, who presented at 2.5 years of age, had a p.W78X mutation and he was diagnosed to have pinealoma and was a case of trilateral RB. Another proband, presented with bilateral disease at 1.5 years with a positive family history. The father had regressed tumour, both the daughter and father carried the same familial mutation, c.1789C > T (Q597Ter). Interestingly, the half sibling of this proband, who was of the same father, presented at the age of 2.3 years with BLRB and had the same mutation c.1789 C > T (Q597Ter). Another interesting aspect was the varied clinical spectrum presentation and outcome of our four BLRB probands who all had the same termination mutation c.1333C > T (p.R445Ter) (Fig. 2).

## Discussion

In the current era of cancer-care—NGS/MLPA techniques have revolutionised the genetic diagnostic scenario of RB-care globally and also selectively in India [22–26]. Incorporating genetic testing as part of RB-care has significant advantages—these opportunities and challenges are highlighted in the current study. For example, our four ULRB cases who would have otherwise not been monitored closely post treatment completion with the mutation, were switched to 3–6 monthly surveillance, like any other BLRB patient with an RB1 mutation in our study cohort. Genetic test as a prognostic marker has been applied in medulloblastoma, paediatric gliomas [55, 56] and breast cancer [56]. However, in comparison, clinical adoption of RB genetic diagnostics is poor amongst the clinicians in India and other developing countries.

The mutation detection rates across countries in BLRB varied from 100 to 16.6% and in ULRB from 56.3 to 9.5% (Table 5), the wide variation could be due to various reasons inclusive of the fact that the studies were performed prior to highly sensitive NGS/MLPA tests era. Price et al., in United Kingdom studied 403 unrelated patients, 209 blood and 194 tumour samples and identified 533 variations, including RB1 gene mutations [39]. In Netherlands, 529 RB patients were screened with a 92% detection rate in BLRB and 10% in ULRB [27]. In the largest mutation meta-analysis of 932 RB patients, it was found that globally the most frequent mutations reported were R320X (nearly 50 times), R579X (nearly 40 times) and R251X (nearly 30 times) [57]. All the studies uniformly found deletions, duplications, missense, nonsense, splice and frameshift mutations, once again establishing that *RB1* gene has no hotspot (10–13, 16, 17, 20–24, 26, 30–32, 34–36, 59–62). In our study, we found 20 *RB1* gene variations in 29 probands (79%), inclusive of three novel mutations, 16 previously reported mutations, four heterozygous deletions of the whole *RB1* gene. We had one case each of frameshift and commonly reported R251X, R320X mutations and it is to be noted that those with the arginine/termination mutations have a risk to develop SPT [15]. In our study, we identified mutations in 86% of BLRB patients and 19% in ULRB—which is comparable to other global studies, however we could not find any mutation in 4 BLRB patients and this could be because of various reasons including mosaicism and somatic *MYCN* gene mutations, which we did not study. Mosaicism is a tricky issue in RB diagnostics and prenatal genetic counselling, hence may go unnoticed suggests Rushlow et al. [58–62].

RB1 gross alterations were found in 15% of 433 BLRB and 6.5% of 262 ULRB patients—these patients developed fewer tumours compared to those with null mutations and interestingly, those with cytogenetic or sub-microscopic whole gene deletions often had ULRB, however all those with gross deletions with one breakpoint inside the RB1 gene had BLRB [63]. Notably, in our cohort all cases of ULRB, irrespective of their mutation type, had optic nerve invasion and were severe enough to warrant enucleation. Prior knowledge of mutation may influence enucleation decisions in the subset of ULRB patients, who all had the mutation, the other eye is also ‘at risk’ and must be treated potentially as a ‘bilateral’ case. In the four c.1333C > T (p.R445Ter) BLRB patients, three had disease progression despite treatment, in one bilateral globe salvage was successful by using plaque brachytherapy, two needed unilateral enucleation and one case needed bilateral enucleation due to progressive disease unresponsive to multimodality treatment (Fig. 2). The variable clinical phenotype and response to treatment despite the same mutation, could be due to epigenetic molecular events in the tumor [64]. In pineal cyst, a pre-malignant form of pinealoblastoma, BLRB is more common than ULRB where germline mutations are invariably identified [65] and we had a patient with pinealoma, trilateral RB who had the pW78X mutation.

In our study the mutation group had statistically significant progression, recurrence and higher percentage of optic nerve invasion, subretinal seeds and high-risk pathological factors but lower percentage of enucleation compared to the non-mutation group. Radiotherapy is contraindicated in patients with germline mutations and this valuable information could help the clinician to modify treatment options. There are studies describing ill effects of radiation on RB, which however do not have the mutation data [66].

Testing the RB1 gene for mutation is a challenging task, owing to its size, heterogeneity of mutations (with 200 reported), lack of hotspot and the variable intronic lengths [67]. Three patients in our cohort were exclusively referred for mutation analysis from other centres, envisaging the fact that clinical management of RB is well addressed, however the same level of care does not exist for genetic testing uniformly across RB care in India. This is despite established RB guidelines specifying the role of genetic testing in RB care [8]. Centres for RB care without a genetic support, must be aware of this need and should sensitize the family on the role and usefulness of genetic testing and also inform them of the additional cost of care to the family which is usually not covered by insurance (68).

There are many limitations of our study, we did not analyse tumor DNA samples and hence we did not detect somatic mutations especially in non-hereditary retinoblastoma [37]. Also, the conclusions made in the study were based on not performing chromosomal studies for large deletion, the study also had a small sample size, with short follow-up period and failure to detect mutations in few BLRB patients. In addition, the ULRB cases had a very low detection rate compared to other robust similar studies.

## Conclusion

In summary, 50 RB patients were screened for *RB1* mutations using targeted NGS and MLPA methodologies, which found detection rates on par with most global studies. Comparing case-wise genetic findings with various clinical parameters and mutations found that there were clinical phenotypic and allelic heterogeneities. The mutation group had a higher clinical risk of recurrence, which influenced clinical management. *RB1* mutation screening is an important tool in RB-care globally, including developing countries.

## Data Availability

The sequencing datasets generated and analyzed during the current study are available in the NCBI Sequence Read Archive (SRA) repository (https://www.ncbi.nlm.nih.gov/bioproject/PRJNA725658) under the BioProject ID PRJNA725658. The ClinVar IDs for the three RB1 variants: NM_000321.3(RB1):c.10508_10502del,NM_000321.3(RB1):c.1331A > C(p.Gln444Pro),NM_000321.3(RB1):c.1699 T > C(p.Ser567Po)are SCV001571344.1,SCV001571345.1,SCV001571346.1 respectively. Further informations on the current study are available from corresponding author on reasonable request.

## Abbreviations

RB: Retinoblastoma
RB1: Retinoblastoma gene
NGS: Next generation sequencing
MLPA: Multiplex ligation dependent probe amplification
ULRB: Unilateral retinoblastoma
BLRB: Bilateral retinoblastoma
MDR: Mutation detection rate
AJCC: American Joint Committee on Cancer
TNM: Tumor node metastasis
DNA: Deoxyribo nucleic acid
OMIM: Online Mendelian inheritance in man
GWAS: Genome Wide Association Study
HGMD: Human gene mutation database
SPSS: Statistical package for the social sciences
LOVD: Leiden open variation database
BWA: Burrows wheeler aligner
EDTA: Ethylenediaminetetraacetic acid
LOH: Loss of heterozygosity
SPT: Secondary primary tumors
CAP: Certified analytics professional
